# Analysis of the effect of sentiment analysis on extracting adverse drug reactions from tweets and forum posts

**DOI:** 10.1016/j.jbi.2016.06.007

**Published:** 2016-08

**Authors:** Ioannis Korkontzelos, Azadeh Nikfarjam, Matthew Shardlow, Abeed Sarker, Sophia Ananiadou, Graciela H. Gonzalez

**Affiliations:** a National Centre for Text Mining, School of Computer Science, University of Manchester, Manchester Institute of Biotechnology, 131 Princess Street, M1 7DN Manchester, United Kingdom; b Department of Biomedical Informatics, Arizona State University, Mayo Clinic, Samuel C. Johnson Research Building, 13212 East Shea Boulevard, Scottsdale, AZ 85259, United States

**Keywords:** Adverse drug reactions, Social media, Sentiment analysis, Text mining

## Abstract

**Objective:**

The abundance of text available in social media and health related forums along with the rich expression of public opinion have recently attracted the interest of the public health community to use these sources for pharmacovigilance. Based on the intuition that patients post about *Adverse Drug Reactions (ADRs)* expressing negative sentiments, we investigate the effect of sentiment analysis features in locating *ADR* mentions.

**Methods:**

We enrich the feature space of a state-of-the-art *ADR* identification method with sentiment analysis features. Using a corpus of posts from the DailyStrength forum and tweets annotated for *ADR* and indication mentions, we evaluate the extent to which sentiment analysis features help in locating *ADR* mentions and distinguishing them from indication mentions.

**Results:**

Evaluation results show that sentiment analysis features marginally improve *ADR* identification in tweets and health related forum posts. Adding sentiment analysis features achieved a statistically significant *F*-measure increase from 72.14% to 73.22% in the Twitter part of an existing corpus using its original train/test split. Using stratified 10 × 10-fold cross-validation, statistically significant *F*-measure increases were shown in the DailyStrength part of the corpus, from 79.57% to 80.14%, and in the Twitter part of the corpus, from 66.91% to 69.16%. Moreover, sentiment analysis features are shown to reduce the number of *ADRs* being recognized as indications.

**Conclusion:**

This study shows that adding sentiment analysis features can marginally improve the performance of even a state-of-the-art ADR identification method. This improvement can be of use to pharmacovigilance practice, due to the rapidly increasing popularity of social media and health forums.

## Highlights

• Sentiment analysis features are useful in spotting adverse drug reactions in text.
• Sentiment analysis features help to distinguish adverse drug reactions and indications.
• Posts about adverse drug reactions are associated with negative feelings.

## Graphical abstract

**Figure:**
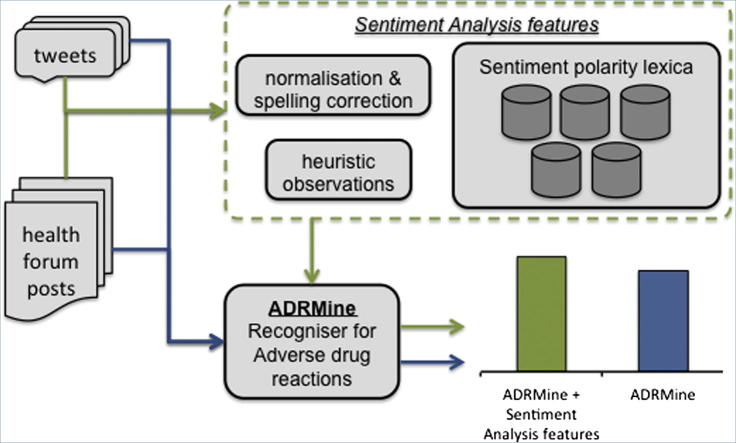

## 1 Introduction

*Adverse Drug Reactions (ADRs)* are among major public health concerns and one of the leading causes of morbidity and mortality [1]. Although the efficiency and safety of drugs are tested during clinical trials, many ADRs remain undiscovered and may only be revealed under specific conditions such as: after long-term use, when used in conjunction with other drugs, or when used by patients who were excluded from the trials such as adults with other morbidities, children, the elderly or pregnant women. Post-marketing drug safety surveillance is therefore necessary to monitor the safety of drugs after approval. *Spontaneous reporting systems (SRS)* are monitoring mechanisms established and supported by regulatory agencies such as the Food and Drug Administration in the United States. These surveillance mechanisms enable both patients and healthcare providers to report suspected ADRs. However, it is estimated that more than 90% of the ADRs still remain unreported, showing the limited effectiveness of SRS [2].

In the United Kingdom, two large resources of medical records, *The Health Improvement Network (THIN)* database and the *General Practice Research Database (GPRD)*, have been used broadly for extracting ADRs [3]. *THIN* contains medical and prescription records, as well as personal information, e.g., date-of-birth and gender, for millions of patients. *GPRD* consists of approximately 4.8 million anonymized UK patient records, collected from 600 general practices, since 1987. *THIN* and *GPRD* exhibit some level of overlap [4]. In addition, data suitable for pharmacovigilance are being generated through the *Yellow Card Scheme*,^2^ an *SRS* available from the *Medicines and Healthcare products Regulatory Agency (MHRA)*. The scheme provides a platform for reporting ADRs directly.

Social media, such as *Twitter* and specialized health-related social networks such as *DailyStrength*,^3^ have provided a relatively new platform enabling patients and care-givers to share and seek information about drug safety. Drug-related posts in social media are a potential source to augment current pharmacovigilance efforts [5]. Although a few individual experiences may not be interesting, thousands of posts about ADRs can potentially minimize unnecessary harmful and sometimes fatal effects.

Pharmacovigilance from social media data is a recent research topic and has undergone significant progress over the last two years. A pioneering study focused on DailyStrength posts regarding six drugs, of which 3600 were manually annotated for ADR mentions [6]. 450 annotated comments were used for developing a lexicon and the remaining for evaluation (*F*-measure 0.74). Following this study, a number of ADR extraction approaches have been proposed for social media based pharmacovigilance [5], [7], [8], [9], [10], [11]. They can be broadly categorized into lexicon-based and non-lexicon-based approaches, with the majority of approaches falling in the former category. Due to the popularity of lexicon-based approaches, various resources containing common ADR assertive terms/phrases have been developed, such as the *Consumer Health Vocabulary (CHV)* [9], a database for mapping words and phrases representing ADRs from lay persons to technical terms used by health professionals. Since the resource contains terms and phrases used by non-experts, it has become popular for pharmacovigilance research. Recent studies focus on combining lexicons for ADR extraction [8]. Lexicon-based approaches face a number of obstacles when applied to social media data. In social media, users rarely use technical terms. Instead, they use creative phrases, descriptive symptom explanations, and idiomatic expressions, the majority of which are not available in existing lexicons. Social media posts frequently contain phonetic and typographic misspellings, which cannot be handled by lexicon-based approaches. Mainly due to these disadvantages of lexicon-based approaches, recent work has focused on using alternatives, based on patterns [7] and supervised learning. Our recent work [5] explored the use of Conditional Random Fields (CRF) for extracting ADRs from social network posts originating from Twitter and DailyStrength. A detailed review of ADR extraction systems can be found in a recent survey [12]. In a separate study, we explored the use of user sentiment indicating features for classifying ADR assertive user posts [13]. Our experiments revealed that users tend to express negative sentiments when mentioning ADRs, and thus, sentiment features contribute in improving classification accuracies. In this study, we focus on utilizing various sentiment-indicating features for the more advanced task of extraction (rather than simply classification) of ADR mentions, and report on our findings.

Sentiment analysis is the process of measuring automatically the type of opinion, i.e. positive, negative or neutral, expressed in text. For example, tweets A and B in Table 1 express positive and negative sentiment, respectively, and tweet C expresses neutral sentiment, since it presents a fact. Some tweets are not easy to identify as positive, negative or neutral. For example, Tweet D contains the word perfect, which indicates positive sentiment, however the tweet is negative, because it is ironic. Tweet E starts with a positive sentence, but the second sentence cancels it ironically.

The first approaches for sentiment analysis matched textual units with opinion words in lexica previously annotated for sentiment polarity [14], [15], [16]. Sentiment lexica list words, *n*-grams and non-contiguous pairs of *n*-grams scored for sentiment. Manually constructed lexica are smaller than automatically constructed ones, due to manual annotation costs [17], [18], [19]. Lexica can be constructed automatically by using seeds of sentiment-conveying words, locating their occurrences in large collections of documents, e.g., the web [20], and scoring words or phrases that co-occur with the seed ones. Similarly, sentiment lexica can be constructed automatically using Twitter. Tweets are annotated based on evidence of positive or negative sentiment they contain, such as emoticons or the hashtags #perfect, #wonderful, #bad and #terrible, to name a few. Then, all words in annotated tweets are scored according to the number of positive and negative tweets in which they occur [21], [22]. As tweets D and E indicate, knowing the sentiment polarity of single words does not guarantee correctly synthesizing the sentiment in a tweet. Considering domain adaptation [14], [15], syntax and various forms of negation is crucial [23]. Machine-learning classifiers have been employed to combine knowledge bases with text characteristics that correlate with sentiment [24], [25]. Latest reviews of the sentiment analysis field focus on summarizing methods and applications of sentiment analysis in the last decade [26], [27], [28]. Sentiment analysis has been employed for a wide variety of applications: social media and blog posts, news articles in general or with respect to a specific domain such as the stock market, reviews of various products, services and shops, emails, stories, narratives, biographies novels and fairy tales.

Automatic analysis of patient posts have received attention in the last few years as a direct source that can help in understanding patients, enhancing the quality of care and increase patient satisfaction. Twitter messages have been classified according to sentiment to estimate how concerned Twitter users are about disease outbreaks [29]. Sentiment analysis has been applied on patient comments posted at the *English National Health Service (NHS)* website in 2010 to compute opinion about various performance aspects in hospitals [30]. Sentiment analysis has also been used on health forum posts of the *Health* & *Wellness Yahoo! Groups* to suggest drugs to be included in *FDA’s Watchlist* [31]. Social media posts have been analyzed to measure changes in sentiment strength in relation to PM2.5 air pollution events in China [32]. The correlation between mortality rates from *atherosclerotic heart disease (AHD)* and behavioral and psychological health factors reflected by tweets from the same region have been investigated [33]. A regression model based on tweets was shown to be able to predict *AHD* significantly better than a model based on demographic, socioeconomic and health risk factors. In the most relevant study to this paper, sentiment in tweets and blog posts was analyzed over time to locate ADRs [34]. The proposed approach was shown to be able to detect ADRs earlier and with higher recall than other methods. However, in that study, posts were not annotated for ADRs, and analysis aims to locate messages that might reflect ADRs. In contrast, the context of the present work is to locate specific ADR mentions in a single post or tweet, in order to aggregate the information obtained to uncover potential signals of ADRs in the population at large.

In this paper, we analyze sentiment in ADR mentions from online user posts about drugs. Our hypothesis is that patient sentiments related to potential ADRs are predominantly negative, and are expressed in online threads of medical social media [35]. We expect mentions of indications, i.e. the conditions targeted by the medication, and beneficial effects or unexpected positive effects, to be accompanied with words and phrases that express neutral or positive sentiments. Let us consider the following tweet including an expression of an indication and of an ADR:

*well my [****Effexor****]*_*Drug name*_*kinda did its job to keep my [****anxiety****]*_*Indication*_*under control…. but now to get my [****heart rate****]*_*ADR*_*down….. ugh*

Negative feelings are expressed about the ADR, *heart rate*, and the medication, *Effexor*, through an informal exclamation, i.e. *ugh*. The indication, anxiety, is excluded from these negative feelings because of the contrasting conjunction, but, and the phrases did its job and under control, which show that the drug affected them beneficially.

Based on this hypothesis, we address the task of locating ADR mentions using heuristics and precompiled knowledge to measure the strength of positive and negative sentiment expressed in social media posts. In particular, we add features used in sentiment analysis systems to ADRMine, our best approach for extracting ADR mentions, introduced in our previous work [5]. Although ADRMine is an existing state-of-the-art system, enriching it with sentiment features is an important discovery step. The innovation lies neither in the existing tool, nor in the sentiment analysis features, but in the novel combination of both. To the best of our knowledge, this is the first study attempting to quantify the effect of sentiment analysis in identifying ADR mentions. We evaluate our approach by measuring whether enriching the system with various types of features related to sentiment analysis leads to a better performance than the original system in locating the exact lexical sequences that express ADRs. We analyze the results to measure whether the enriched system can better distinguish between ADR and indication mentions. Evaluation results show that sentiment analysis features marginally improve ADR identification in tweets and posts in forums related to public health. Moreover, they are shown to reduce the number of ADRs being recognized as indications.

## 2 Methods

In this section we discuss details about the manually derived corpus of *Adverse Drug Reactions (ADRs)* and indication mention annotations, the baseline system, *ADRMine*, and the sentiment analysis features that we integrated into it. The corpus used for experimentation in this paper has been used previously for ADR extraction in [5].

### 2.1 Corpus

We use posts from *DailyStrength* and *Twitter*, associated with 81 drugs. For both sources, the first step in our data collection process involved the identification of a set of drugs to study, followed by the collection of user comments associated with each drug name. Details about the choice of drugs have been discussed in our past publications [5], [10], [12], [36]. Each post was annotated by two expert annotators independently. The annotations include medical concepts belonging to the categories: ADR, beneficial effect, indication, and other. Each annotation includes the span of the mention with start/end position offsets, the semantic category, the drug name, and the corresponding *UMLS (Unified Medical Language System)* concept ID. We measured inter-annotator agreement using *Cohen’s kappa* [37] for both data sets. The calculated kappa value for approximate matching of the annotated concepts is 0.85 for DailyStrength and 0.81 for Twitter. The values can be considered to indicate high agreement. For this study, we use a subset of the two corpora by including only those posts for which there were complete agreements between the two annotators. We only include ADR, indication and beneficial effect mentions, and considered the beneficial effects as indications due to their similarity. The Twitter corpus consists of 1782 tweets, while the DailyStrength corpus consists of 6279 user posts. Both corpora were divided in a training and test part according to a 75/25 ratio. For Twitter, we use 1339 instances for training and 443 for testing; for DailyStrength we use 4720 for training and 1559 for testing. Table 2 shows corpora statistics.

### 2.2 Baseline system

To evaluate the correlation between ADR occurrences and sentiment expressed in social media text, we add sentiment analysis features to an existing system for extracting ADR mentions, which we summarize here. Different types of sentiment analysis features are added separately to demonstrate their effect on the result. ADRMine [5] is a supervised sequence labeling *Conditional Random Field (CRF)* classifier. It is trained on annotated mentions of ADRs and indications, and classifies individual tokens in sentences. Individual sentence tokens are the classification candidates. ADRMine uses the IOB (Inside, Outside, Beginning) scheme for encoding the concept’s boundaries. It learns to distinguish 5 different labels: *B-ADR*, *I-ADR*, *B-Indication*, *I-Indication* and *Out*. The feature set used to represent classification instances consists of the following feature types:

• ***Context features***: Seven features representing the context, i.e., the current ±3 tokens in the sentence. The tokens are lemmatized into WordNet roots using the Dragon toolkit [38] and the spelling errors are corrected using the Apache Lucene^4^ spell checker library. More information is available in [5].
• ***ADR lexicon-based feature***: A binary feature that shows whether the current token exists in the ADR lexicon. The ADR lexicon contains a list of ADRs and the associated UMLS IDs collected from different resources [5].
• ***Parts-of-Speech***: Part of speech of the token, generated using Stanford parser.
• ***Negation***: A feature indicating whether the token is negated in the sentence or not. Negations are identified by considering grammatical dependency relations between negation words (e.g., no, not, any and less) and the target token. For instance consider the sentence: “*It had no improving effect*”. *Effect* is considered as negated since there is a dependency relation that indicates negation between effect and no (neg(effect, no)). We also consider a token negated if it is occurs in a window of two tokens after a negation word. For instance, improving in the example sentence is also considered negated [39].
• ***Embedding cluster features***: Considering the characteristics of user posts in social media, often there are several unseen or rarely occurring tokens in the test sentences. ADRMine uses a set of features that represent the semantic similarity between words. In a preprocessing step, the words from a large unlabeled corpus of user posts are divided into 150 clusters where each cluster contains semantically similar words (words that occur in similar contexts are considered semantically similar). The clusters are generated based on word embedding vectors learned by training a language model on more than a million sentences [40]. ADRMine uses the cluster numbers of the current ±3 tokens as embedding cluster features [5].

### 2.3 Sentiment analysis features

For this paper, we add sentiment awareness to ADRMine [5], by enriching it with a variety of features that have been proved to perform well in the latest evaluation tasks of the SemEval series, task 2 in SemEval 2013 [41] and task 9 in SemEval 2014 [42]. Both evaluation tasks defined subtasks about analyzing sentiment at term level, where participating systems were required to estimate the sentiment polarity of a given word in context, or message level, where participating systems had to estimate the sentiment polarity of entire messages. Systems were tested in a number of domains: regular tweets, sarcastic tweets, mobile phone text messages (SMS) and sentences posted to the *LiveJournal* weblog. After inspecting participating systems, we concluded that the majority employed the following information sources in various formats, depending on the architecture of each:

• ***Token n-grams***, i.e., contiguous sequences of tokens: usually, *n* ranges from 1 to 4.
• ***Non-contiguous pairs of token n-grams***, i.e. *n*-grams that occur in the same sentence but not in succession. For example, in the sentence “*It had no improving effect*”, the pair *(it had, improving effect)* is a non-contiguous bigram-bigram pair, while the pair *(it had, effect)* is a non-contiguous bigram-unigram pair. Sentiment analysis systems usually consider unigram-unigram, bigram-bigram, unigram-bigram and bigram-unigram pairs.
• ***Parts-of-speech of tokens***
• ***Character n-grams***, i.e., sequences of contiguous characters in tokens: usually, *n* ranges from 3 to 5.
• ***Observations on the surface forms of tokens***, i.e., capitalization, punctuation, elongated words. Elongated words contain one or two repeating characters more than twice, e.g., *soooo* and *goood*.
• ***Negation indicator***: usually sentiment analysis systems identify negated phrases by considering lists of negation trigger words, e.g. *no*, *none* and *never*, and syntactic analysis.
• ***Token normalization*** usually refers to spelling correction as well as automatic identification of abbreviated tokens and replacement with the corresponding full forms. Abbreviations are particularly common in Twitter, due to the restriction in message length.
• ***Sentiment polarity lexica***, i.e., lists of words, phrases or non-contiguous sequences of words with associations to positive or negative sentiments.

Token surface forms and token surface form *n*-grams are useful to quantify sentiment in trainable machine learners, based on the principle that similar texts possibly express similar sentiment polarity. *N*-grams are used to capture the immediate context so as to disambiguate the meaning of the current token, since different senses of a token might be related to different polarities. Similarly, the parts-of-speech of tokens as well as character subsequences of tokens are considered to be informative for sentiment analysis. Non-contiguous pairs of token *n*-grams are useful to capture distant dependencies in text that correlate with sentiment polarities.

In online posts, capital letters are used to indicate anger or emphasis of content. Moreover, elongated words and punctuation symbols, such as exclamation and question marks are used to designate emotions. Repeated punctuation can express extra emotional strength. To capture correlation with sentiment, we use capitalization, elongated words and punctuation as information sources.

Negated phrases are important when measuring sentiment, because the polarities expressed in them are negated. For example, in the phrase “this is nice!”, “nice” bears positive sentiment, while in the phrase “this is not nice!” it bears negative sentiment. We considered sequences that start with a negation word and end in a punctuation mark [43], i.e., , : ; ! ?. The list of negation words in Christopher Potts’ sentiment tutorial was used.

Due to the length restriction of tweets and typing errors, text in social media exhibits higher variability than text of other domains, such as scientific publications. Increased variability leads to sparsity, when processing text with machine learning tools. To reduce sparsity in the space of words we used Twitter Word Clusters (TW clusters) [44], a set of 1000 clusters of similarly spelled words. While developing of a part-of-speech tagger for tweets, these clusters were produced by applying the Brown clustering algorithm on 56 million tweets in English. The clusters contain 216,856 distinct words. Mapping the vast number of correctly spelled or misspelled words to a significantly smaller set of cluster ids, creates links between frequent and less frequent words, respectively, and addresses sparsity.

### 2.4 Lexica

Sentiment polarity lexica are lists of words, *n*-grams and non-contiguous pairs of words scored according to the sentiment load they carry. We used five popular lexica: the *Hu*&*Liu Lexicon of Opinion Words (H*&*L)* [17], the *Subjectivity Lexicon (SL)* [18], the *NRC Word-Emotion Association Lexicon (NRC)* [19], the *NRC Hashtag Sentiment Lexicon (NRC#)* [20], and the *Sentiment 140 Lexicon (S140)* [22]. The first three were developed manually, while the last two were automatically constructed.

*H*&*L* contains approximately 6800 words and was developed manually from e-commerce customer reviews. It is formatted as an alphabetically sorted word list that consists of a positive part (29.55%, 2006 entries) and a negative part (70.45%, 4783 entries). Entries are not lemmatized or associated with positive or negative scores. Thus, all positive entries are considered equally positive and all negative words are considered equally negative.

*SL* contains 8222 single-word entries, compiled from a number of manually or automatically created resources from annotated and un-annotated data as part of OpinionFinder [18]. Each entry word is annotated to express weak or strong subjectivity and accompanied with its part-of-speech and a positive (35,62%, 2718 entries) or negative (64,38%, 4913 entries) polarity label. *NRC*contains approximately 14,000 alphabetically ordered words manually annotated on a set of tweets using *Amazon Mechanical Turk*. Apart from positive and negative general sentiment, the words were also annotated for eight basic emotional dimensions, i.e. anger, fear, sadness, disgust, surprise, anticipation, trust, and joy, following *Plutchik’s Theory of Emotion* [45].

*NRC#* is developed automatically from 775,310 tweets, posted between April and December 2012. Automatic annotation is based on hashtags that indicate sentiment, such as #good, #excellent, #bad and #terrible. 78 sentiment-bearing seed words were used to classify tweets as positive or negative. Each word w occurring in a positive (*p*) or negative (*n*) tweet is scored according to the formula: PMI(w,p)-PMI(w,n), where PMI stands for Pointwise Mutual Information. Positive and negative scores indicate respective sentiment and the magnitude indicates association strength. Apart from the list of unigrams (54K entries), the lexicon also contains a similarly created list of bigrams (317K entries), and lists for non-contiguous pairs of unigrams and bigrams (309K entries) that occurred in a single tweet.

*S140* is developed similarly to *NRC*. Its development was based on a corpus of 1.6 million tweets that contain positive and negative emoticons. It provides entries for unigrams, bigrams, and non-contiguous pairs of unigrams and bigrams, i.e. unigram-unigram, bigram-bigram, unigram-bigram and bigram-unigram pairs.

### 2.5 Applying sentiment analysis features to ADR extraction

The task of predicting ADR location in text is different than both subtasks of the SemEval tasks discussed above. In the first subtask the target is to quantify the sentiment polarity of a specific word in a message. Thus, participants addressed each message as a single instance. Similarly, in the second subtask, where the target is to quantify the sentiment expressed in entire messages, each message is again a single instance. To predict the exact position of ADR mentions, each message token needs to be encoded as a separate data-mining instance. Consequently, the transformation of information sources into discriminative features was performed differently than in SemEval participating systems. For example, the SemEval participating system *NRC-Canada* [21] used the sentiment scores for each token to compute message level features such as the sum of sentiment scores for all tokens. Moreover, while for the SemEval tasks *n*-gram frequencies were considered, token frequencies do not apply to the current task. In the task at hand, information sources were transformed into the following set of features per token and were used to train and test ADRMine [5]:

• ***token n-grams***: seven features encoding the surface forms of the current ±3 tokens.
• ***lemmas***: seven features encoding the lemmas of the current ±3 tokens.
• ***parts-of-speech***: seven features encoding the parts-of-speech of the current ±3 tokens.
• ***isAllCaps***: a feature denoting if the token is in capital letters.
• ***isPunctuation***: a feature denoting if the token consists of questionmarks (?) and/or exclamation marks (!).
• ***isElongated***: a feature denoting whether the token contains a repeating letter, e.g. soooo.
• ***isNegated***: a feature denoting whether the token is part of a negated sequence, according to the definition of negation discussed in section Sentiment analysis features.
• ***token weights in lexica***: five features encoding the sentiment polarities assigned to the current token in each of the five lexicon: *H*&*L*, *SL*, *NRC*, *NRC#* and *S140*.
• ***bigram and non-contiguous n-gram pair weights in lexica***: eight features encoding the sum of sentiment polarities assigned to bigrams and non-contiguous unigram and bigram pairs, in which the current token participates. Two lexica were considered for these features: *NRC#* and *S140*.
• ***drug name*** & ***minimum sentiment relative position***: a binary feature denoting whether the minimum sentiment token precedes or follows the drug name in a message (if there is a drug name mention). The feature is based on the observation that the position of drug names affects ADR mention identification.
• ***character n-grams***: three features encoding all 3-, 4- and 5-grams in the current token and their frequencies.
• ***TW clusters***: seven features encoding the numbers of TW clusters that contain the current ±3 tokens, if the tokens are included in some TW cluster or 0, otherwise.

Although some of these features are not directly related to sentiment, e.g. parts-of-speech, they are considered in this study, because they are features of state-of-the-art sentiment analysis systems. As expected, some features types in sentiment analysis systems have been used in our baseline system, ADRMine (Section 2.2). In particular the original ADRMine feature set also includes lemmas, parts-of-speech and negation. We choose to evaluate these features in the experiments of the current study, since they were captured using methods different than the ones used in the original feature set of ADRMine. Each feature type was evaluated separately, so as to quantify its contribution.

The sentiment analysis lexica were used to compute weights of lexical units. Weights for each token were computed independently for each lexicon and were considered as independent features. In *H*&*L* and *NRC*, words are annotated as positive or negative. Thus, we considered unary weights. *SL* contains positive and negative annotated words for either strong or weak subjectivity. To take subjectivity annotations into account we adopted unary weights for strong subjectivity annotations and weights of 1/2 for weak subjectivity annotations. The automatically annotated corpora, i.e., *NRC#* and *S140*, contain weighted positive and negative annotation for unigrams, bigrams and non-contiguous *n*-gram pairs. We used these weights directly for computing feature values.

ADRMine enriched with features of sentiment analysis systems, as explained above, is evaluated against the original ADRMine model [5]. Systems are compared as far as their ability to locate the exact lexical sequences that express ADRs. We investigate whether sentiment analysis features help to distinguish between indications mentions and ADR mentions and whether sentiment analysis features can help in locating posts that contain ADRs.

## 3 Results

Due to the large number of features (17 baseline and 49 sentiment analysis features) we have merged them in feature groups for experimentation. Evaluating each group of features separately, allows to measure its contribution. Table 3 shows which features in the previous section are grouped together. Features drawn using each of the five lexica comprise a separate group. In particular, we experiment with using the features drawn by each lexicon separately and all lexicon features together (see experiment *All Lex.*). We also evaluate collectively all features of sentiment analysis systems (see experiment *All SA features*).

Since the task is to identify ADRs, we investigated the hypothesis that the positions of drug name occurrences in text are correlated with the associated ADR mentions. To evaluate it we included a binary feature indicating whether the closest drug name occurs before or after the minimum sentiment position in a sentence (see feature *DN* & *min. sent. pos.*). Minimum sentiment positions were computed by averaging all sentiment lexica scores per sentence token. The *DN* & *min. sent. pos.* feature was evaluated separately and in conjunction with all features of sentiment analysis systems (see experiment *All features*).

As a baseline, we have used ADRMine [5]. In all experiments, ADRMine’s feature set was extended by adding each feature group in Table 3. To compare against gold-standard annotations (predicted mentions), typical information retrieval evaluation measures are employed using approximate matching. An extracted mention is considered as matching a gold-standard mention if it is contains the gold-standard mention. For example, the mention “*serious bone problems*” is counted as matching if “*serious bone problems*” or “*bone problems*” are gold-standard mentions. Precision (*P*), recall (*R*) and *F* measure ($F_{1}$) are computed as follows:

(1) $$P=\frac{\left|\mathrm{matching}\;\mathrm{mentions}\right|}{\left|\mathrm{predicted}\;\mathrm{mentions}\right|}$$

(2) $$R=\frac{\left|\mathrm{matching}\;\mathrm{mentions}\right|}{\left|\mathrm{actual}\;\mathrm{mentions}\right|}$$

(3) $$F_{1}=\frac{2\times P\times R}{P+R}$$

Table 4 shows the preliminary evaluation results^5^ of the baseline and the new feature sets (Table 3).

**Fig. 1:**
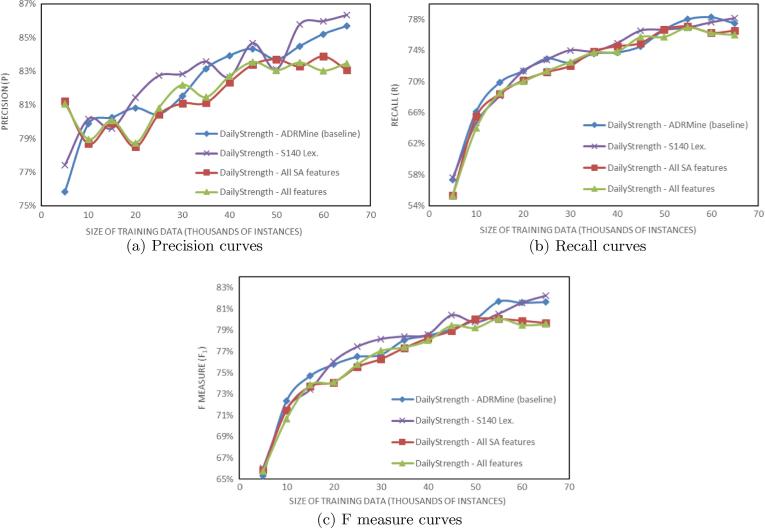
Evaluation for the DailyStrength part of the corpus using parts of the training data.

**Fig. 2:**
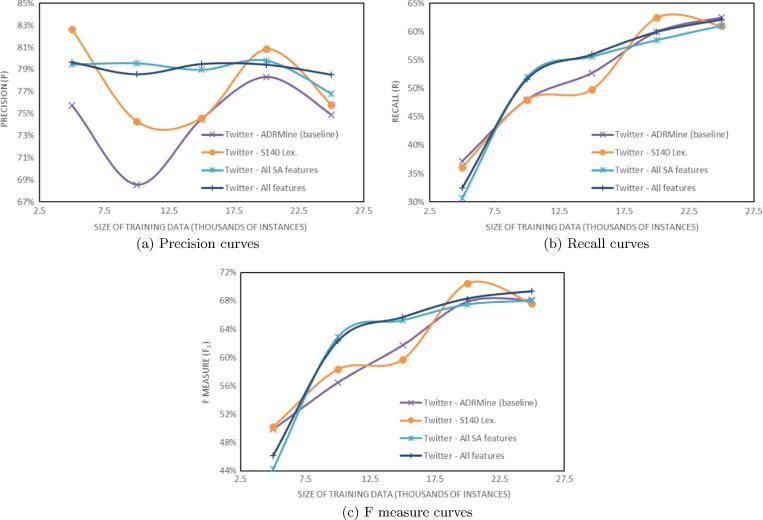
Evaluation for the Twitter part of the corpus using parts of the training data.

To investigate the effect of the size of the training data on the results, we have trained the *ADRMine baseline* and the three best performing systems, i.e. *S140 Lex.*, *All SA features* and *All features* (Table 4) using increments of 5K training instances. The results are plotted in Fig. 1 for the DailyStrength part of the corpus and in Fig. 2 for the Twitter part of the corpus.

In order to further investigate the statistical significance of the improvements in Table 4, we conducted a stratified 10×10-fold cross validation experiment. We merged the training and test data instances and allocated them randomly to 10 folds, making sure that all folds have an approximately equal number of ADR and indication mentions. We repeated 10 times the random allocation to 10 folds, using different randomisation seeds. We repeated each experiment in Table 4 10 times for each of the 10 random allocations, each time training on 9 folds and testing on 1-fold. Table 5 shows the average results over these 100 experiments per feature group as well as statistical significance information.

Table 6 shows our evaluation at mention level using the original training and test split. The first three columns describe how ADR mentions were predicted by the baseline system and the best performing feature set, for each corpus. Similarly, columns 4–6 show prediction numbers and percentages for mentions that were manually annotated as indications. Columns 7 and 8 show false positives, i.e. the numbers of mentions that were predicted wrongly, because there were no matching mentions annotated manually.

Table 7, Table 8 display evaluation at the message level. Table 7 shows how messages that only contain ADRs or indication mentions were predicted by the baselines and the best performing feature sets. Table 8 shows results for messages that contain both types of mentions and no mentions at all.

## 4 Discussion

The results of our main experiment, in Table 4, show that the heuristics feature set and the feature set containing all proposed features (Table 3) have a modest but significant impact on the ADRMine baseline performance for the Twitter corpus. In the DailyStrength corpus, no statistically significant increase or decrease was observed using any of the proposed feature sets, while the feature set encoding the Sentiment 140 Lexicon performed best. Interestingly, this feature set also achieves the highest precision in the Twitter corpus. It should be noted, that ADRMine is already a sophisticated system, incorporating features about many aspects of ADR mentions. Thus, performance increase by adding more information sources is expected to be small.

One reason why sentiment features improved ADR recognition for Twitter but not for DailyStrength may lie in the differences of message characteristics posted in these social media. Intuitively, the differences in the size of datasets and the different percentages of ADR mentions that they contain (see Table 2) may also be a reason. To investigate this further we conducted a series of experiments by incrementally increasing the size of training data, as shown in Fig. 1, Fig. 2. The plots reveal that all evaluation metrics depend on the size of training data. However, plots for Twitter data are much lower than the respective plots for DailyStrength.

Due to the message length restriction of tweets, authors write elliptically, with grammar, syntax and spelling errors, while in DailyStrength no such restrictions apply. DailyStrength is a specialized forum for health, thus participants feel more confident to discuss details about their medication. Indicatively, ADRMine already achieves very high *F*_1_ score (>80%) in identifying ADRs in DailyStrength posts and sentiment analysis features cannot contribute significantly. In contrast, sentiment analysis features can aid ADR recognition significantly in tweets, which are shorter and more difficult to analyze.

The stratified 10×10-fold cross validation results, in Table 5 reveal more statistically significant results for both corpora. The feature sets that combine features types, i.e. *All SA features* and *All features*, achieved the highest *F* measure scores, both in Twitter and DailyStrength. It is worth noting that the margin of improvement in our approach is small due to our strong baseline, ADRMine, which is a state-of-the-art system for identifying ADR mentions. Experimental results show that the improvement achieved by integrating sentiment analysis features is statistical significant to a high degree, confirming our intuition that sentiment features are helpful in the identification of ADR mentions.

Table 5 also indicates the contribution of each feature set to the final result for each part of the corpus. For DailyStrength messages, the most meaningful features are token and lemma *n*-grams, part-of-speech tags and token-character *n*-grams. For identifying ADRs in tweets, the most meaningful features are token-character *n*-grams, features induced from sentiment lexica, TW cluster features and token and lemma *n*-grams. We observe that for each part of the corpus each feature set contributes differently, due to the characteristics of the feature set and the messages in that part. For example, TW cluster features are expected to be more useful on tweets, since TW clusters were generated using a very large corpus of tweets [44].

Looking at how gold-standard ADR mentions are identified by the best performing methods in comparison to the baselines (6 first columns in Table 6) no particular improvement can be observed. However, the last two columns reveal that sentiment-aware methods succeed in reducing ADR false positives significantly (10–18%). In DailyStrength, sentiment-aware features predicted less ADR mentions as indications. An equal number of ADR mentions were classified as normal text. This effect cannot be captured by the standard information retrieval evaluation metrics. However, if a system cannot recognize some ADRs correctly, it is better to classify them as normal text than as mentions of another type, as confusing the class could negatively affect the performance of downstream applications, e.g., identifying indications or specific signals of potential ADRs for pharmacovigilance.

The message-level evaluation (Table 7, Table 8) confirms that the best performing sentiment-aware feature sets improve ADR extraction, to a limited extent. Confirming our results at the mention level, the fifth column of Table 8 shows that the sentiment-aware feature set classifies more messages that contain no mentions as such. For tweets, we observe no increase in correctly classified messages that contain no mentions, despite the outcome of the mention-level evaluation (seventh column, Table 6), because the particular messages contain more than one mention.

Apart from the evaluation results shown in the previous section, we computed prediction accuracies for different classes of messages separately, considering whether the drug name discussed in the message is mentioned once or more times, or it is not mentioned. Further, we investigated separately the messages in which the drug name is mentioned before or after the ADR mention. The main observation in this analysis is that in both corpora, ADR mentions are predicted more accurately (76–78%) in cases where a drug name mention precedes them, than in cases where a drug name mention follows them (67–72%). Adding a binary feature to our best performing setting (All SA features) to encode whether a token is (part of) a drug name, increased performance on tweets but not on DailyStrength posts, as shown in Table 9. This was expected, as drug names are rarely mentioned in DailyStrength posts, due to the nature of the website, where users post under specific treatments (drug names). Interestingly, accuracy in predicting indication mentions is not affected by drug name mentions occurring before or after them.

Analyzing mentions that were not recognized correctly, we identified three major classes of error. Firstly, some messages contained words and sequences that indicate positive sentiment such as “loved” in example A, in Table 10 and “very well” in example B. Although the proposed features consider negated phrases, it is likely that the occurrence of positive words and sequences fool the CRF classifier. Moreover, examples A and B contain ADR mentions that are particularly difficult to recognize because they are periphrastic. Secondly, ADRs in ironic messages, such as example C, cannot be predicted correctly, because words and expressions typically associated with positive sentiments are used to express negative feelings. Thirdly, too general ADRs, such as the ADRs in examples D and E in Table 10, were particularly difficult to recognize. Furthermore, we observed that the CRF classifier rarely predicts ADRs whose mentions contain an embedded drug name mention, such as in example D.

Inspired by this error analysis, in the future we plan to investigate further how we can identify ADRs expressed periphrastically. In addition, we plan to incorporate syntactical features. Due to the size of the current corpus, we expect to encounter severe sparsity effects, thus capturing syntax will probably be attempted in conjunction with methods for sparsity reduction, such as using reference corpora. We consider this study as one of the first steps towards assessing whether social media can contribute positively in detecting ADRs. In the future, we plan to compare and contrast ADRs extracted from the social media with ADRs extracted using more traditional methods.

## 5 Conclusion

Social media and health-related forums comprise important resources for pharmacovigilance. Due to the size of data available, automatic identification of *Adverse Drug Reaction (ADR)* mentions is crucial. In this paper, we hypothesized that, in online posts, *ADR* mentions are associated with negative sentiment. We investigated this hypothesis by enriching *ADRMine*, a state-of-the-art system for extracting *ADR* mentions, with sentiment-aware features. We evaluated our approach on a collection of tweets and DailyStrength posts that were manually annotated for *ADR* and *indication* mentions. Evaluation results showed that sentiment-bearing features marginally improve *ADR* mention identification in tweets and health-related forum messages. In addition, the proposed approach was shown to disambiguate *ADRs* and *indication* mentions better than the best configuration of the baseline system, *ADRMine*.

## Conflict of interest

None.

## Figures and Tables

**Table 1:** Examples of tweets about drugs.

#	Example tweet
A	*I only have like 3 days left of my [****Trazodone****]*_*Drug* *name*_*and*
	*I don’t have refills GREEAAATTT!*
B	*[****Cymbalta****]*_*Drug* *name*_*, my mood has worsened*
C	*Depression hurts. [****Cymbalta****]*_*Drug* *name*_*can help.*
D	*hello, world! [****Quetiapine****]*_*Drug* *name*_*zombie this morning and I have a work call in about 45 minutes. Perfect combination.*
E	*I think I like this [****Seroquel****]*_*Drug* *name*_*stuff. Pity I have to think.*

**Table 2:** Numbers of ADR and indication mentions in the DailyStrength and Twitter corpora, number of messages and numbers of messages depending on the mentions they contain. Percentages (%) are shown within parentheses.

Corpus	Mentions		Messages	Messages containing			
ADR	Ind.	ADRs	Ind.	Both	None
*Training*							
Daily-Strength	1500	1068	4720	1500	1068	232	2384
(31.8)	(22.6)	(4.9)	(50.5)
Twitter	651	101	1339	651	101	53	640
(48.6)	(7.5)	(4.0)	(47.8)

*Test*							
Daily-Strength	752	454	1559	533	322	71	775
(34.2)	(20.7)	(4.6)	(49.7)
Twitter	277	38	443	236	33	18	192
(53.3)	(7.5)	(4.1)	(43.3)

**Table 3:** Feature groups used for experimentation in this section.

Feature groups	Types of included features
*n*-grams	Token *n*-grams, lemmas
PoS	Parts-of-speech
Character *n*-grams	Character *n*-grams
Negation	isNegated
Heuristics	isAllCaps, isPunctuation, isElongated
TW	TW clusters
Lex.	Token, bigram and non-contiguous *n*-gram pair weights in lexica
All SA features	*n*-grams, PoS, character *n*-grams, negation, heuristics, TW & Lex. features for all lexica
DN & min. sent. pos.	Drug name & minimum sentiment relative position
All features	All SA features, DN & min. sent. pos.

**Table 4:** ADR extraction performance percentages (on DailyStrength and Twitter) when testing different feature sets.

	DailyStrength			Twitter		
Features	*P*	*R*	*F*_1_	*P*	*R*	*F*_1_
ADRMine (baseline)	86.34	78.40	82.18	76.51	68.23	72.14
*n*-grams	86.25	76.93	81.32	74.38	64.98	69.36
Character *n*-grams	85.40	77.20	81.09	78.70	65.34	71.40
PoS	85.02	77.20^∗^	80.92	75.95	64.98	70.04
Negation	86.38	78.67	82.34	76.35	66.43	71.04
Heuristics	86.41	78.00	81.99	76.92	68.59	72.52
TW	85.55	78.13	81.67	74.49	65.34	69.62
Hu&Liu Lex.	86.26	77.87	81.85	77.05	67.87	72.17
Subjectivity Lex.	85.86	77.73	81.60	75.61	67.15	71.13
NRC Lex.	86.32	78.27	82.10	74.27	64.62	69.11
NRC# Lex.	85.74	76.13	80.65	76.09	63.18	69.03
S140 Lex.	**87.19**	**78.93**	**82.86**	79.48	65.70	71.94
All Lex.	86.38	76.93	81.38	78.30	66.43	71.88
All SA features	83.82	77.33	80.44	**77.89**^∗^	**68.59**^∗^	**72.94**^∗^
DN & min. sent. pos.	86.39	77.87	81.91	74.60	66.79	70.48
All features	83.36	77.13	79.58	**78.51**^∗^	**68.59**^∗^	**73.22**^∗^

***Note***: The contents of each feature-set are presented in Table 3. Statistically significant improvements over the baseline are marked with asterisk (^∗^). Statistical significance was computed using the two-tailed *McNemar’s Q* for a confidence level of 0.05.

**Table 5:** ADR extraction performance percentages (on DailyStrength and Twitter) when testing different feature sets. Stratified 10 × 10-fold cross-validation results.

	DailyStrength			Twitter		
Features	*P*	*R*	*F*_1_	*P*	*R*	*F*_1_
ADRMine (baseline)	83.62	75.96	79.57	75.51	60.29	66.91
*n*-grams	83.91	76.55^‡^	80.03^‡^	75.85	60.32	67.07
Character *n*-grams	83.07	76.96^‡^	79.87^∗^	76.05	62.32^‡^	68.39^‡^
PoS	83.31	76.81^‡^	79.89^‡^	74.30	60.36	66.47^∗^
Negation	83.71	75.93	79.59	75.35	60.16	66.77
Heuristics	83.67	76.03	79.62	75.58	60.29	66.94
TW	83.21	76.71^‡^	79.80	75.90	60.99^†^	67.50^†^
Hu&Liu Lex.	83.55	76.10	79.62	75.66	60.46	67.08
Subjectivity Lex.	83.58	76.01	79.58	75.61	60.53	67.09
NRC Lex.	83.46	75.98	79.51	75.49	60.60	67.10
NRC# Lex.	83.47	75.56	79.29	75.92	60.92^∗^	67.48^∗^
S140 Lex.	83.48	76.03	79.55	75.78	60.84^∗^	67.37^∗^
All Lex.	83.22	75.98	79.40	76.04	61.50^‡^	67.88^‡^
All SA features	83.04	**77.50**^‡^	**80.14**^‡^	**77.02**^‡^	**62.98**^‡^	**69.18**^‡^
DN & min. sent. pos.	83.63	75.97	79.58	75.44	60.38	66.95
All features	83.01	**77.51**^‡^	**80.14**^‡^	76.90	**63.01**^‡^	**69.15**^‡^

***Note***: Statistically significant improvements over the baseline are marked with asterisk (∗), dagger (†) and doubledagger (‡) for significance levels of 0.05, 0.01, 0.005, respectively. Since the cross-validation folds are common between all experiments, the two-tailed matched-samples *t*-test was used for computing statistical significance.

**Table 6:** Prediction numbers and (within parentheses) percentages of ADR or indication mentions in DailyStrength (DS) and Twitter messages by the baseline system and the best performing systems for each corpus.

	ADR mentions			Indication mentions			No mentions	
Features	Predicted as			Predicted as			Predicted as	
ADR	Ind.	None	Ind.	ADR	None	ADR	Ind.
*DS*								
ADRMine	598	38	116	306	45	103	44	35
(baseline)	(79.5)	(5.1)	(15.4)	(67.4)	(9.9)	(22.7)
S140 Lex.	597	34	121	305	44	105	40	30
(79.4)	(4.5)	(16.1)	(67.2)	(9.7)	(23.1)	(−10)	(−14)

*Twitter*								
ADRMine	189	0	88	13	6	19	50	1
(baseline)	(68.2)	(0.0)	(31.8)	(34.2)	(15.8)	(50.0)
All features	192	0	85	15	7	16	41	4
(69.3)	(0.0)	(30.7)	(39.5)	(18.4)	(42.1)	(−18)	(+300)

***Note***: For unannotated text (last two columns) parentheses shows increase or decrease in comparison to the relevant baseline.

**Table 7:** Prediction numbers and (within parentheses) percentages of DailyStrength (DS) and Twitter messages that contain ADR or indication mentions by the baseline system and the best performing systems for corpus.

	Messages containing ADR mentions				Messages containing indication mentions			
Features	Predicted as containing				Predicted as containing			
ADRs	Ind.	Both	None	Ind.	ADRs	Both	None
*DS*								
ADRMine	442	22	67	69	178	101	66	43
(baseline)	(82.9)	(4.1)	(12.6)	(13.0)	(55.3)	(31.4)	(20.5)	(13.4)
S140 Lex.	444	22	69	67	177	101	70	44
(83.3)	(4.1)	(13.0)	(12.6)	(55.0)	(31.4)	(21.7)	(13.7)

*Twitter*								
ADRMine	176	3	6	57	7	16	6	10
(baseline)	(74.6)	(1.3)	(2.5)	(24.2)	(21.2)	(48.5)	(18.2)	(30.3)
All features	178	4	9	54	6	17	9	10
(75.4)	(1.7)	(3.8)	(22.9)	(18.2)	(51.5)	(27.3)	(30.3)

**Table 8:** Prediction numbers and (within parentheses) percentages of DailyStrength (DS) and Twitter messages that contain both ADR or Indication mentions or no mentions at all by the baseline system and the best performing systems for each corpus.

	Messages containing ADR and indication mentions				Messages containing no mentions			
Features	Predicted as containing				Predicted as containing			
Both	ADRs	Ind.	None	None	ADRs	Ind.	Both
*DS*								
ADRMine	48	17	6	0	748	18	9	0
(baseline)	(67.6)	(23.9)	(8.5)	(0.0)	(96.5)	(2.3)	(1.2)	(0.0)
S140 Lex.	52	13	6	0	756	12	7	0
(73.2)	(18.3)	(8.5)	(0.0)	(97.6)	(1.6)	(0.9)	(0.0)

*Twitter*								
ADRMine	6	7	3	2	170	21	1	0
(baseline)	(33.3)	(38.9)	(16.7)	(11.1)	(88.5)	(10.9)	(0.5)	(0.0)
All features	8	5	3	2	173	17	2	0
(44.4)	(27.8)	(16.7)	(11.1)	(90.1)	(8.9)	(1.0)	(0.0)

**Table 9:** ADR extraction performance (on DailyStrength and Twitter) when testing the *isDrugName feature.*

	DailyStrength			Twitter		
Features	*P*	*R*	*F*_1_	*P*	*R*	*F*_1_
All SA features + isDrugName	83.24	76.80	79.89	**78.13**^∗^	**68.68**^∗^	**73.66**^∗^
All features + isDrugName	83.79	77.20	80.36	**77.37**^∗^	**67.87**^∗^	**72.31**^∗^

***Note***: Statistically significant improvements over the baseline are marked with asterisk (∗). Statistical significance was computed using the two-tailed *McNemars Q* for a 95% confidence interval.

**Table 10:** Examples of messages whose ADR mentions were not predicted correctly.

#	Example
A	*loved it, except for [****not being able to be woken up****]*_*ADR*_*at night* …
*yeah that blew*

B	*Worked VERY well at first.*
*Now it is [****hard to tell I am taking it****]*_*ADR*_*at all.*
*Almost afraid to get off.*

C	*just woke up from a [****14 hour nap****]*_*ADR*_*thank u [****Fluoxetine****]*_*Drug* *name*_

D	*@thatjunkiechick thank you!*
	*[****coming off****[****Effexor****]*_*Drug* *name*_***is NOT fun****]*_*ADR*_*!*

E	*Seemed to help at the beginning, but quickly [****lost effectiveness****]*_*ADR*_
*and side effects got bad.*

## Footnotes

2 http://yellowcard.mhra.gov.uk. Accessed on November 10, 2015.

3 http://www.dailystrength.org. Accessed on November 10, 2015.

4 http://lucene.apache.org. Accessed on November 10, 2015.

5 The table shows micro-average results, i.e. results at corpus level. This method was preferred against macro-average, i.e. computing results at message level, because the vast majority of messages contain zero or one ADR mention, only.
